# Improved Consistency of Lung Nodule Categorization in CT Scans with Heterogeneous Slice Thickness by Deep Learning-Based 3D Super-Resolution

**DOI:** 10.3390/diagnostics15010050

**Published:** 2024-12-28

**Authors:** Dongok Kim, Jae Hyung Park, Chang Hyun Lee, Young-Ju Kim, Jong Hyo Kim

**Affiliations:** 1 Department of Applied Bioengineering, Graduate School of Convergence Science and Technology, Seoul National University, Seoul 08826, Republic of Korea; dongk@snu.ac.kr; 2 ClariPi Research, Seoul 03088, Republic of Korea; 3 Department of Radiology, Seoul National University Hospital and College of Medicine, Seoul 03080, Republic of Korea; parkjh4803@gmail.com (J.H.P.); changhyun.lee@snu.ac.kr (C.H.L.); 4 Division of Imaging Medical Device Research, Department of Medical Device Innovation Research, Seoul National University Hospital, Seoul 03080, Republic of Korea; 21762@snuh.org; 5 Center for Medical-IT Convergence Technology Research, Advanced Institutes of Convergence Technology, Suwon-si 16229, Republic of Korea

**Keywords:** computed tomography, deep learning, super-resolution, slice thickness

## Abstract

**Background/Objectives**: Accurate volumetric assessment of lung nodules is an essential element of low-dose lung cancer screening programs. Current guidance recommends applying specific thresholds to measured nodule volume to make the following clinical decisions. In reality, however, CT scans often have heterogeneous slice thickness which is known to adversely impact the accuracy of nodule volume assessment. **Methods**: In this study, a deep learning (DL)-based 3D super-resolution method is proposed for generating thin-slice CT images from heterogeneous thick-slice CT images in lung cancer screening. We evaluated the performance in a qualitative way by radiologist’s perceptual assessment as well as in a quantitative way by accuracy of nodule volume measurements and agreement of volume-based Lung-RADS nodule category. **Results**: A 5-point Likert scale tabulated by two radiologists showed that the quality of DL-generated thin-slice images from thick-slice CT images were on a par with the image quality of ground truth thin-slice CT images. Furthermore, thick- and thin-slice CT images had a nodule volume difference of 52.2 percent on average which was reduced to a 15.7 percent difference with DL-generated thin-slice CT. In addition, the proposed method increased the agreement of lung nodule categorization using Lung-RADS by 74 percent. **Conclusions**: The proposed DL approach for slice thickness normalization has a potential for improving the accuracy of lung nodule volumetry and facilitating more reliable early lung nodule detection.

## 1. Introduction

Recently, the ‘Strengthening the screening of Lung Cancer in Europe’ (SOLACE) project has been established which has sparked a significant increase in chest CT examinations across Europe. However, various CT scanners represent CT images with heterogeneous slice thickness, and this discrepancy leads to significant challenges in accurate volumetric measurements of the lung nodules, as it is known that slice thickness is inversely proportional to the precision in lung nodule volumetric measurements [1]. According to the Lung CT Screening Reporting and Data System version 2022 (Lung-RADS v2022) established by the American College of Radiology, lung nodules with volumes of 113 mm^3^ or greater have an increased risk of developing into tumors [2,3]. Therefore, the precise measurement of lung nodule volume is critical for early detection and intervention before its progression to malignancy.

Recent advances in deep learning, particularly in super-resolution techniques, have shown notable improvements over traditional interpolation methods in image quality restoration. Dong et al. [4] introduced one of the first super-resolution methods utilizing a three-layer convolutional neural network (CNN), outperforming classic interpolation methods. However, the shallow depth of layers limited its ability to faithfully restore high-frequency components in images. Building on this, Kim et al. [5,6] applied ideas from ResNet, using a 32-layer CNN with skip connections to address the vanishing gradient problem. The network with larger parameters enabled the restoration of high-frequency details. Zhang et al. [7] further enhanced the network by introducing a residual-in-residual structure, achieving high peak signal-to-noise ratio (PSNR) and structural similarity index measure (SSIM).

Ledig et al. [8] observed that relying solely on L1 loss functions resulted in blurry outputs, as they favor averaged pixel values and thus sacrifice high-frequency details. Moreover, PSNR and SSIM metrics do not always directly reflect perceived image quality. To address this, they introduced perceptual loss derived from pre-trained VGG network feature maps and employed a generative adversarial network (GAN) for training. In this framework, the generator produces super-resolved (SR) images from low-resolution (LR) inputs, while the discriminator attempts to distinguish between the SR images and high-resolution (HR) ground truths. Through adversarial training, the generator learns to produce images indistinguishable from HR images, a network they named SRGAN. Despite not achieving the highest PSNR and SSIM values, SRGAN produced images that were perceptually closer to HR images.

Following this, the enhanced SRGAN (ESRGAN) was developed by Wang et al. [9], who deepened the network using residual-in-residual dense blocks without batch normalization, further improving image quality. Subsequently, Real-ESRGAN [10] introduced additional degradations such as noise, blur, and JPEG compression during training, making the network more robust for real-world images.

In the medical imaging domain, researchers have actively adapted deep learning techniques to advance the field. Park et al. [11,12,13] utilized residual networks to upsample in the z-direction and generate thin CT slices, leveraging this technology to explore radiomic features in chest CT. Yun et al. [14,15] generated thin CT slices to achieve higher quality orbital bone reconstructions. Other studies have employed slice generation for visualizing spine structures and early-stage lung adenocarcinomas [16,17,18].

Given these advancements, we propose a DL-based 3D super-resolution method to generate thin-slice CT images from heterogeneous thick-slice inputs to improve consistency of lung nodule categorization.

The following are the main contributions of this paper:

- To the best of our knowledge, modified ESRGAN to take in 3D inputs such as CT images has not been used to observe lung nodules by generating thin slices from thick-slice CT.
- We show that slice generation on thick-slice CT images are essential for retaining lung nodule texture, accurate lung nodule volumetric measurement, and the corresponding categorization of lung nodules based on Lung-RADS v2022.

By enhancing the resolution in the depth direction, our approach improves the accuracy of lung nodule measurements and thus results in consistent lung nodule categorization. This will facilitate the more reliable, early detection of lung nodules, thereby supporting the process of lung cancer screening programs.

## 2. Materials and Methods

### 2.1. Training Dataset

For training the deep learning model, we utilized CT image data from the National Lung Screening Trial (NLST) open dataset. From hundreds of thousands of series, a total of 28,628 chest CT images with a slice thickness of 1.0 mm were selected. Among these images, 18,243 were acquired using Siemens Sensation 16 scanners (Erlangen, Germany), 2931 from Canon Aquilion scanners (Otawara, Japan), 2707 from GE LightSpeed 16 scanners (Chicago, IL, USA), and 4747 from Philips MX8000 scanners (Amsterdam, Netherlands) as shown in Table 1.

### 2.2. Test Dataset

**Figure 1:**
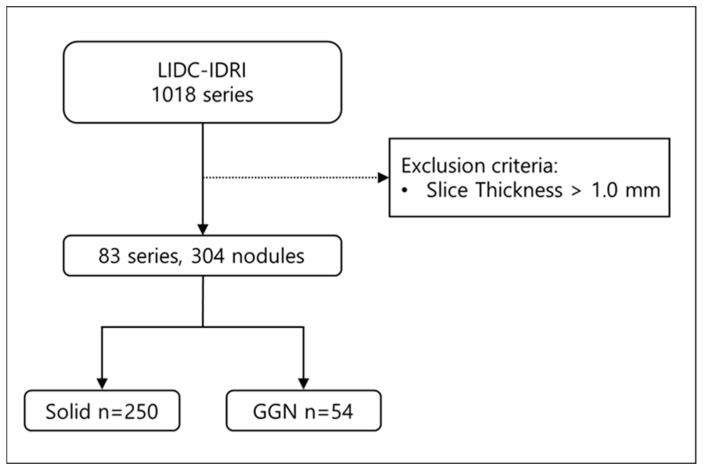
Flow diagram of lung nodule inclusion for analyzing the model performance qualitatively and quantitatively.

To evaluate the performance of the model, we employed the Lung Image Database Consortium and Image Database Resource Initiative (LIDC-IDRI) open dataset. LIDC-IDRI consists of diagnostic and lung cancer screening thoracic CT scans with annotated lung nodules. The dataset contains 1018 thoracic CT scans; among them, 55 scans had a slice thickness of 1.0 mm, and 28 scans had a slice thickness of 0.75 mm. We included CT scans with a slice thickness of 1.0 mm or less in this study to enable direct comparison of lung nodule characteristics between thick-slice, generated thin-slice, and thin-slice CT scans. From a total of 83 CT scans, 304 lung nodules were identified and manually segmented (Figure 1), from which all of them were used for qualitative and quantitative analysis.

### 2.3. Model Architecture

We selected ESRGAN as the baseline model for our super-resolution method, making necessary modifications to adapt it for medical imaging. Unlike real-world images, CT image acquisition occurs under controlled environments, ensuring consistent image quality. Therefore, introducing degradations such as noise, blur, and JPEG compression, as performed in Real-ESRGAN [9], was deemed unnecessary for our application.

Traditional super-resolution networks focus on single-image super-resolution. However, CT images are volumetric, necessitating modifications to handle three-dimensional data. We adapted the convolutions in the network to accept three-dimensional arrays, such as 512 × 512 × 16 volumes. Due to GPU memory limitations, CT images were partitioned into segments of 16 slices before being input into the network.

**Figure 2:**
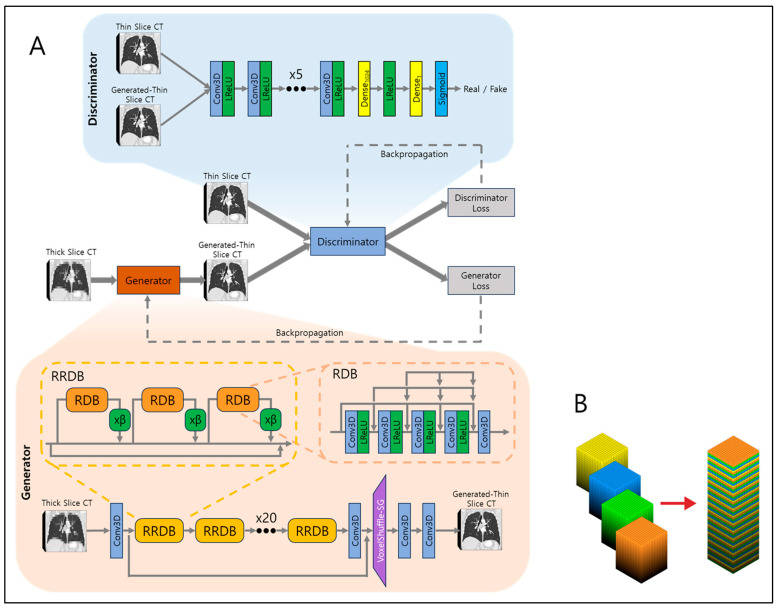
(**A**) Model architecture and (**B**) schematic diagram of VoxelShuffle-SG.

The network architecture is illustrated in Figure 2A. At its core is the generator network, responsible for converting low-resolution (LR) inputs to super-resolved (SR) outputs. The generator employs 23 residual-in-residual dense blocks (RRDBs) as its fundamental building units. Each RRDB has a single skip connection running through the center and comprises three residual dense blocks (RDBs), each multiplied by β which is a residual scaling parameter, set to 0.2. RDB consists of alternating five 3D CNNs and four leaky rectified linear units (LReLUs), and in front of every 3D CNN, dense connections are present to allow residual flow. This design enhances the efficient flow of information and improves the network’s ability to learn complex features. Local residual connections within each RDB further stabilize the training process by mitigating the vanishing gradient problem, commonly encountered in deep networks. The global residual connection links the input of the first RDB to the output of the last RDB, creating a residual-in-residual structure. This dual-level residual design—both within each RDB and across the entire stack of RDBs—enhances the model’s capacity to capture and synthesize high-frequency details essential for producing high-resolution images with fine textures and sharp edges.

After processing through the RRDBs, the network applies another 3D CNN, followed by VoxelShuffle-Slice Generation (SG) module, depicted in Figure 2B, to increase spatial resolution in the depth direction while preserving learned features. The VoxelShuffle-SG is a custom-made module which closely resembles the PixelShuffle module from PyTorch [19], adapted for processing three-dimensional data. While PixelShuffle transfers tensors from the channel domain to the spatial domain in two dimensions, VoxelShuffle-SG extends this functionality to three-dimensional arrays, enabling tensor transfer from the channel domain to the depth dimension. For example, when input tensor of shape (batch, channel × r^2^, height, width) enters PixelShuffle, the tensors are transferred from channel to dimension so that output tensor is of shape (batch, channel, height × r, width × r), where r represents scaling number. Similarly, when input tensor of shape (batch, channel × r, height, width, depth) enters VoxelShuffle-SG, the tensors are transferred from channel to depth so that output tensor is of shape (batch, channel, height, width, depth × r). Finally, two 3D CNNs are applied to output generated thin-slice CT images.

The output is then fed into the discriminator network along with the ground truth thin-slice CT images. The discriminator’s role is to discern generated thin-slice CT images (i.e., fake) and ground truth thin-slice CT images (i.e., real). Its network is composed of eight 3D CNN and LReLU pairs, one dense fully connected layer which outputs 1024 tensors, followed by another LReLU and another dense fully connected layer which output one tensor, and finally a sigmoid function. The output of one would represent the discriminator that assumes the input was ground truth thin-slice CT images and the output of zero would represent the discriminator that assumes the input was generated thin-slice CT images. The discriminator is trained adversarially against the generator, encouraging the generator to produce generated thin-slice CT images increasingly indistinguishable from the ground truth thin-slice CT images. In the ideal case, at the end of model training, the generator would produce generated thin-slice CT images which are perceived as almost identical to the ground truth thin-slice CT images, such that the discriminator cannot discern between the two.

### 2.4. CT Image Pre-Processing

**Figure 3:**
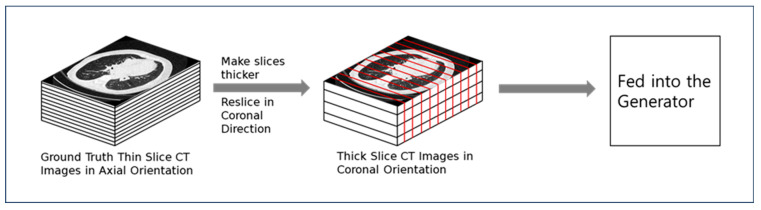
Pre-processing procedure of CT images prior to model processing.

To generate thin slices, CT images reconstructed in the axial plane were resliced into the coronal plane. This re-slicing allows the network to effectively utilize volumetric information by employing three-dimensional convolutions. Due to GPU memory constraints, only 16 slices of coronal images were input into the network at a time. The network outputs coronal images with four times more voxels in the depth dimension, effectively increasing the resolution. Subsequently, the coronal images are resliced back into the axial plane, resulting in axial images with slices that are four times thinner than the original thick slices. This process is illustrated in Figure 3.

### 2.5. Training

For training, we used 23,816 chest CT images with a slice thickness of 1.0 mm. On average, each series contained approximately 300 slices. To simulate thick-slice CT images, every four consecutive slices were averaged to produce a single slice, reducing the number of slices from 300 to 75 per series and increasing the slice thickness from 1.0 mm to 4.0 mm. These low-resolution thick-slice CT images were input into the network to produce generated thin-slice images. The network was trained with the Adam optimizer by setting β_1_ = 0, β_2_ = 0.9, and learning rate of 1 × 10^−4^. It was implemented in Python 3.11 and PyTorch 2.4.0 with four Nvidia GeForce RTX 3090 graphics cards.

The original 1.0 mm thin-slice CT images and the generated 1.0 mm thin-slice images were compared using the following three loss functions: (1) mean squared error (MSE) loss, (2) perceptual loss, and (3) adversarial loss, with equations provided below, as follows:

(1) $$\;l_{n}=\frac{1}{rDHW}\overset{rD}{\underset{z=1}{\sum }}\overset{H}{\underset{y=1}{\sum }}\overset{W}{\underset{x=1}{\sum }}{\left(I_{x,y,z}^{GT}-G_{\theta }{\left(I^{LR}\right)}_{x,y,z}\right)}^{2}$$

(2) $$\;\;l_{VGG}^{SR}=\frac{1}{D_{i,j}H_{i,j}W_{i,j}}\overset{D_{i,\;j}}{\underset{z=1}{\sum }}\overset{H_{i,j}}{\underset{y=1}{\sum }}\overset{W_{i,j}}{\underset{x=1}{\sum }}{\left(\varphi_{i,j}{\left(I^{GT}\right)}_{x,y,z}-\varphi_{i,j}{\left(G_{\theta }\left(I^{LR}\right)\right)}_{x,y,z}\right)}^{2}$$

(3) $$l_{Gen}^{SR}=\overset{N}{\underset{n=1}{\sum }}-logD_{\theta }\left(G_{\theta }\left(I^{LR}\right)\right),$$

where *r* is scaling factor which is 4 for this study; *D* is depth; *H* is height; *W* is width; *I^LR^* is thick-slice CT image; *I^GT^* is the thin-slice CT image; *G_θ_* is the generator function; *D_θ_* is the discriminator function; *φ_i,j_* is the feature map obtained after the *j*-th convolution and before the *i*-th max-pooling layer in the VGG network; and *D_i,j_*, *H_i,j_*, and *W_i,j_* denote the dimensions of the respective feature maps.

The MSE is a pixel-wise metric that measures the average squared difference between the estimated values and the actual values, derived from the square of the Euclidean distance. While minimizing MSE reduces the overall difference in voxel values between predicted and actual images, relying solely on MSE can result in blurry textures due to the suppression of high-frequency details. To address this limitation, we introduced perceptual and adversarial losses.

The perceptual loss for SR was first proposed by Johnson et al. [20] and extended usage in SRGAN and ESRGAN. The perceptual loss employs the pre-trained VGG19 network, a classification model used in the ImageNet dataset challenge. The MSE of the feature map of VGG between SR and HR is calculated and minimized. Since the VGG network is designed for natural two-dimensional images, we could not directly implement it on three-dimensional CT images. Therefore, we split the CT volumes into individual slices and sequentially fed them into the VGG network, calculating the loss by summing and averaging over all slices. By minimizing the Euclidean distance between the feature representations of the ground truth thin-slice CT images and the generated thin-slice CT images, the network produces images that are perceptually similar to each other.

The adversarial loss is derived from the discriminator, which simultaneously encourages the generator to produce images more similar to the ground truth thin-slice images and trains the discriminator to better distinguish between the ground truth and generated images. This adversarial training is carried out until the saturation of loss output, both in the training and validation dataset. The model with completed training fosters the generation of high-quality images that are indistinguishable from the real thin-slice CT images.

### 2.6. Evaluation Methods

For the qualitative evaluation, radiologist 1 and radiologist 2 were given a 5-point Likert scale to score based on the following criteria: visibility of fine lung structures, i.e., how visible are the overall lung structures, nodule margin delineation, i.e., how delineated are the solid nodule margins, and visibility of ground-glass nodule (GGN)/subsolid nodule components, i.e., how visible are the GGN and subsolid nodules. For the generated thin-slice CT images, an additional evaluation assessed the presence of any generated artifacts. This is because since the model architecture is based on GAN, there could be concern regarding fake nodule synthesis, and the evaluation presented the radiologists the choice of responding yes/no regarding the presence of generated artifacts. As for the rest of the evaluation criteria, the quality levels were defined as follows: 1—poor, 2—fair, 3—good, 4—very good, 5—excellent.

For the quantitative analysis, the lung nodules of thick-slice, generated thin-slice, and thin-slice CT images were manually segmented for the volume measurements. The segmentation process involved setting a region of interest (ROI) encompassing the nodule in all three axial, coronal, and sagittal views. The nodule was then separated from surrounding lung structures accordingly. If the nodule was present in only one or two slices in the axial plane, the separation was performed only in that plane. To verify the repeatability of lung nodule volume measurements, a single reference nodule was measured five times, yielding volumes of 133, 121, 146, 142, and 144 mm^3^ for the expert 1 and 137, 140, 133, 125, and 148 mm^3^ for the expert 2. For the expert 1, the average measurement was 137.2 mm^3^ with a standard deviation of 9.23 mm^3^, corresponding to a coefficient of variation of 6.73 percent., and for the expert 2, the average measurement was 136.6 mm^3^ with a standard deviation of 7.61 mm^3^, corresponding to a coefficient of variation of 5.57 percent. Since pixel spacing and slice thickness are known, voxel volume can be calculated, and total nodule volume can be found from the segmentations. A threshold value of −450 was used for separation between nodule types. If all voxels have a CT number above −450, the nodule is considered solid; if all voxels have a CT number equal to or below −450, the nodule is considered GGN; and if voxels have a CT number above and below −450, the nodule is considered sub-solid.

Finally, lung nodule categorization was performed based on the nodule volume measurements and in accordance with categorization criteria of Lung-RADS v2022. where it specifies categories (2—benign, 3—probably benign, 4a—suspicious and 4b—very suspicious), depending on nodule types and volume measurements. The category agreements between the lung nodules of the thick-slice–thin-slice CT pair and generated thin-slice–thin-slice CT pair were derived using confusion matrices where the category agreement of each pair was calculated by summing all the number of nodules present in the diagonal axes and dividing by the total number of nodules.

## 3. Results

### 3.1. Qualitative Analysis

The qualitative evaluation of 304 lung nodules in thick-slice, generated thin-slice and thin-slice CT images using a 5-point Likert scale is shown in Table 2. In all criteria, both radiologists assigned higher scores to the generated thin-slice CT with respect to the thick-slice CT. No artifacts were observed in the generated thin-slice CT.

**Figure 4:**
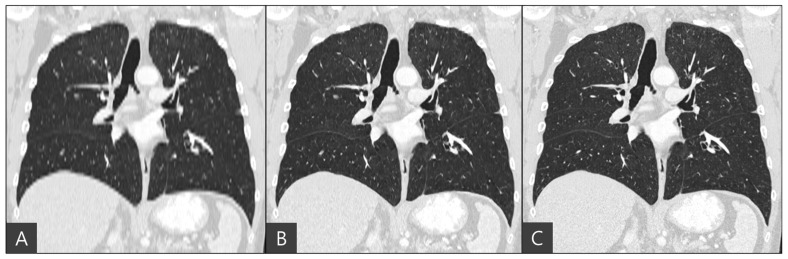
Coronal view of (**A**) thick-slice, (**B**) generated thin-slice and (**C**) thin-slice CT images.

**Figure 5:**
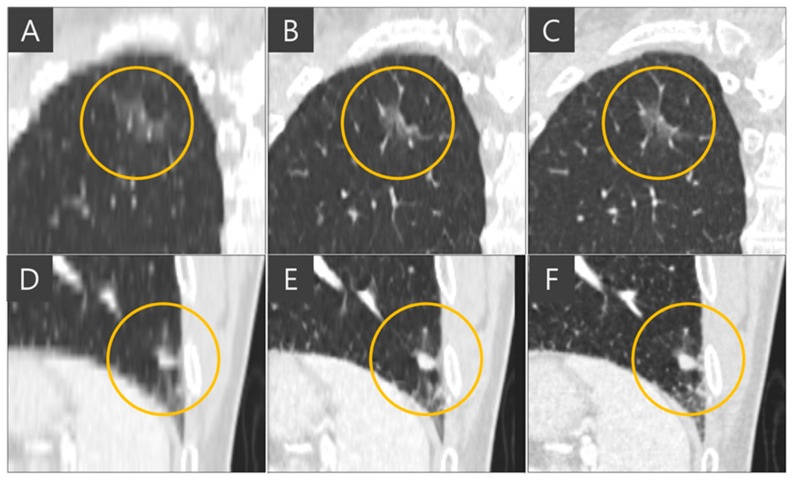
GGN circled in (**A**) thick-slice, (**B**) generated thin-slice, and (**C**) thin-slice CT images and solid nodule circled in (**D**) thick-slice (**E**) generated thin-slice, and (**F**) thin-slice CT images.

The representative coronal views of thick-slice, generated thin-slice, and ground truth thin-slice CT images are presented in Figure 4. The thick-slice CT image exhibits staircase artifacts, unidentifiable fissures, and blurred lung vessels, whereas the generated thin-slice CT image quality is comparable to that of the ground truth thin-slice CT image. Visual inspection of GGN and solid nodules in each image type reveals clearer distinctions. In Figure 5, the GGN in the thick slice CT image has indistinct boundaries, making accurate segmentation and volume estimation challenging. In contrast, the GGN in the generated thin-slice CT image closely resembles that in the ground truth thin-slice CT image, facilitating accurate assessment. The Likert scale scored 1, 4, 5 on the visibility of fine lung structures, 1, 4, 5 on nodule margin delineation, and 1, 4, 5 on visibility of GGN/subsolid nodule component for thick-slice, generated thin-slice, and thin-slice CT, respectively, by both radiologists. Similarly, for solid nodules, the thick-slice CT image lacks sufficient image quality for precise volume measurement, whereas the solid nodule in the generated thin-slice CT image exhibits adequate quality for accurate segmentation, mirroring the ground truth. The Likert scale scored 1, 4, 5 on the visibility of fine lung structures and 1, 4, 5 on nodule margin delineation for the thick-slice, generated thin-slice, and thin-slice CT, respectively, by both radiologists.

**Figure 6:**
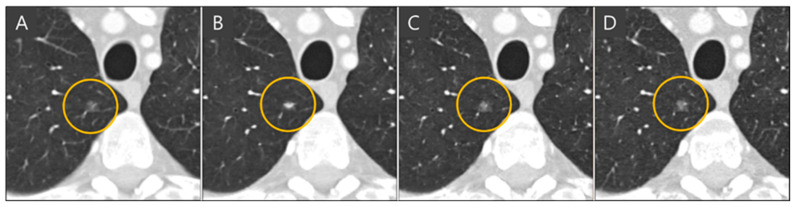
Axial view of (**A**) thick-slice, (**B**) generated thin-slice produced by the model trained on a single slice, (**C**) generated thin-slice produced by the model train on multiple slices, and (**D**) thin-slice CT images, subsolid nodules are circled.

The importance of administration of multiple slices into the network for slice generation is demonstrated in Figure 6. When single slices of coronal CT images are fed into the network, it focuses solely on coronal views and cannot accurately reconstruct images in the sagittal plane, resulting in inaccurate nodule representations when viewed axially. In the ground truth thin-slice CT image and the generated thin-slice CT image produced by the network using sixteen slices simultaneously, a lung vessel passing through a GGN is faithfully reconstructed, preserving both volume and texture. However, the network trained with single-slice inputs failed to accurately reproduce the GGN, resulting in an appearance resembling a subsolid nodule. This highlights the necessity of training the network with multiple slices to capture the full details of three-dimensional CT images.

### 3.2. Quantitative Analysis

Expert 1 and expert 2 each manually segmented 304 lung nodules from thick-slice, generated thin-slice, and thin-slice CT images and the calculated volumes derived from the two segmentations were averaged.

**Figure 7:**
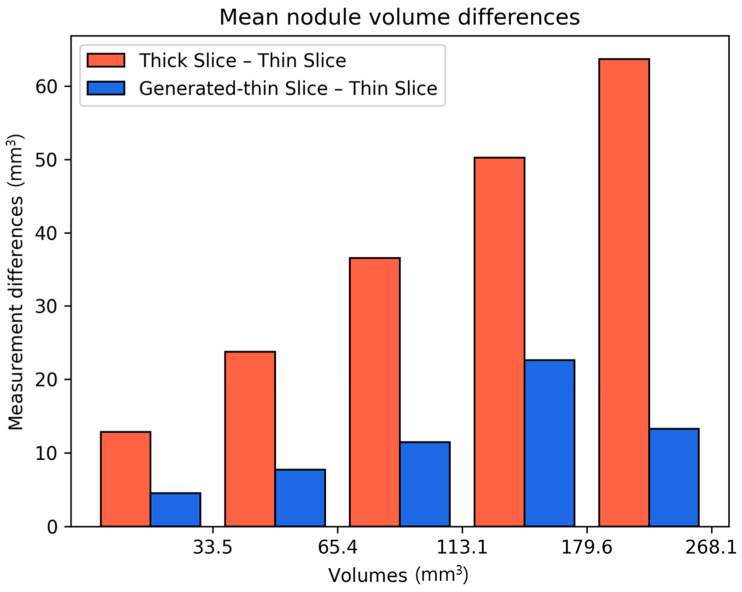
Graph of mean volume error by varying nodule sizes for thick-slice–thin-slice CT pair and generated thin-slice–thin-slice CT pair.

**Figure 8:**
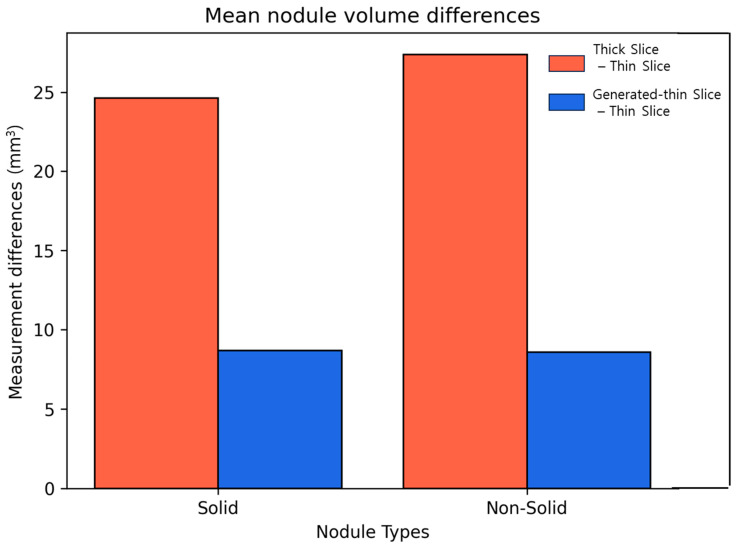
Graph of mean volume error by varying nodule types for thick-slice–thin-slice CT pair and generated thin-slice–thin-slice CT pair.

We calculated the differences in lung nodule volumes between thick-slice and thin-slice CT images, as well as between the generated thin-slice and thin-slice CT images. These differences were categorized by nodule volume (Figure 7) and by nodule types (Figure 8). The volume differences between the generated thin-slice and thin-slice CT images were significantly smaller than those between the thick-slice and thin-slice images in both categorizations.

**Figure 9:**
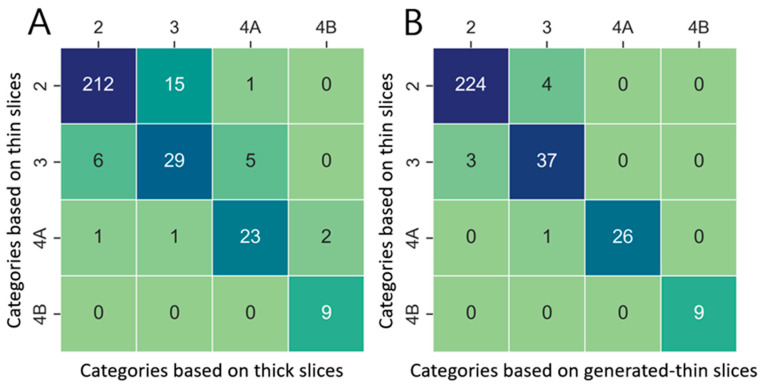
Confusion matrix of Lung-RADS v2022 lung nodule classification for (**A**) thick-slices–thin-slices CT pair and (**B**) generated thin-slices–thin-slices CT pair.

Lung nodule volumes measured in thick-slice, generated thin-slice, and thin-slice CT were categorized according to Lung-RADS v2022. Confusion matrices illustrating the classification outcomes and corresponding categorization definitions are presented in Figure 9 and Table 3, respectively. There were 31 misclassifications between the thick-slice and thin-slice CT, whereas only 8 misclassifications occurred between the generated thin-slice and thin-slice CT. Therefore, the agreement of lung nodule categorization with reference thin-slice CT images increased by 74 percent.

## 4. Discussion

The accurate measurement of the lung nodule volume is critical for proper categorization based on Lung-RADS v2022, particularly for solid nodules around 113 mm ^3^—the threshold between category 2 (benign) and category 3 (probably benign). Our results indicate that lung nodules from thick-slice CT images were misclassified 3.9 times more often than those from generated thin-slice CT images when compared to thin-slice CT images.

Older CT scanners typically produce images with slice thicknesses between 3 and 5 mm. Even with newer generation CT scanners, thick-slice images are often stored in Picture Archiving and Communication Systems (PACS) for storage efficiency. In both scenarios, the slice thickness is inadequate for precise nodule volume measurements, underscoring the necessity for methods to convert thick slices to thin slices.

Our deep learning model, based on ESRGAN, was modified to accommodate 3D CT images by adjusting the input channels and replacing the PixelShuffle module with the VoxelShuffle-SG module. While single-slice generation can recover nodule volume, it fails to accurately restore nodule texture. By extending the model to process three-dimensional data, both nodule volume and texture were faithfully recovered. Due to GPU memory constraints, we limited the input to 16 slices for slice generation. This proved sufficient, as the generated thin-slice CT images exhibited similar image quality to the ground truth thin-slice images, as observed by a radiologist, and demonstrated quantitatively similar volumes upon manual segmentation. This level of accuracy enables reliable lung nodule classification using Lung-RADS v2022, even from thick-slice CT images.

There are limitations to this study. Although training was conducted using data from four major CT vendors (Siemens – Erlangen, Germany, GE – Chicago, IL, USA, Canon – Otawara, Japan, and Philips – Amsterdam, Netherlands), all images were sourced from the NLST open dataset. Similarly, the test data originated from the LIDC-IDRI open dataset. Future work should include validating the model’s performance on CT images from other institutions and with various CT scanners to ensure generalizability.

## 5. Conclusions

Heterogeneous thick-slice CT images introduce inconsistency in the quality of lung nodule volumetric properties which is an essential factor in low-dose cancer screening programs. Therefore, a proper way to neutralize the effect of slice thickness is required, and in this research, we proposed a DL-based super-resolution method to convert heterogeneous slice thickness to thin-slice CT images. The resulting generated thin-slice and reference thin-slice CT image qualities were evaluated qualitatively and quantitatively. The qualitative evaluation was performed by the radiologist’s perceptual assessment using a 5-point Likert scale, and regarding the visibility of fine lung structures, the score increased from 1.28/1.3 to 4.02/3.71 for generated thin-slice CT which is on par with the score of 4.95/4.98 for thin-slice CT. The quantitative evaluation was performed with lung nodule volume measurement and lung nodule categorization. The average nodule volume difference between thick- and thin-slice CT was 52.2 which was reduced to 15.7 percent when compared between generated thin- and thin-slice CT. Lung nodule classification had 74 percent increased agreement between generated thin- and thin-slice CT. In all evaluation tools, generated thin-slice CT showed better performance than original thick-slice CT. In conclusion, when only thick-slice CT images are available, converting them to thin-slice CT images are crucial for reliable lung nodule classification, which would facilitate early lung nodule detection and appropriate follow-up clinical management.

## Figures and Tables

**Table 1:** Parameters of training datasets.

Manufacturer	Siemens Erlangen, Germany	Canon Otawara, Japan	GE Chicago, IL, USA	Philips Amsterdam, Netherlands
Model	Sensation 16	Aquilion	LightSpeed 16	MX8000
Tube Voltage (kV)	120	120	120	120
Slice Thickness (mm)	1.0	1.0	1.0	1.0
Reconstruction Kernel	B30f, B45f, B50f, B80f	FC01, FC02, FC53	Standard, Lung	C
Number of Series	60	11	10	16
Number of Slices	18,243	2931	2707	4747

**Table 2:** The 5-point Likert scale table of qualitative image evaluation by two radiologists.

		Radiologist 1			Radiologist 2	
Categories	Thick Slice	Generated-Thin Slice	Thin Slice	Thick Slice	Generated-Thin Slice	Thin Slice
Visibility of fine lung structures	1.28	4.02	4.95	1.3	3.71	4.98
Nodule margin delineation	1.38	3.98	4.85	1.44	3.65	4.94
Visibility of GGN/Subsolid nodule components	1.67	3.50	4.17	1.71	3.29	4.57
Generated artifacts	N.A.	No artifacts	N.A.	N.A.	No artifacts	N.A.

N.A.—not applicable.

**Table 3:** Simplified Lung-RADS v2022 lung nodule categories.

Lung-RADS	Category	Findings
2	Benign	Solid nodule: <113 mm^3^ Part solid nodule: <113 mm^3^
3	Probably Benign	Solid nodule: ≥113 to <268 mm^3^ Part solid nodule: ≥113 mm^3^ total volume with solid component <113 mm^3^
4A	Suspicious	Solid nodule: ≥268 to <1767 mm^3^ Part solid nodule: ≥113 mm^3^ total volume with solid component≥113 to <268 mm^3^
4B	Very Suspicious	Solid nodule: >1767 mm^3^ Part solid nodule: Solid component ≥268 mm^3^

## Data Availability

The LIDC-IDRI CT image dataset used for the evaluation is available at the following URL: https://www.cancerimagingarchive.net/collection/lidc-idri/ accessed on 10 June 2023. The NLST CT image dataset used for training the model is available at the following URL: https://www.cancerimagingarchive.net/collection/nlst/ accessed on 21 November 2022.

## References

[1] Petrou M., Quint L.E., Nan B., Baker L.H. (2007). Pulmonary Nodule Volumetric Measurement Variability as a Function of CT Slice Thickness and Nodule Morphology. Am. J. Roentgenol..

[2] MacMahon H., Naidich D.P., Goo J.M., Lee K.S., Leung A.N.C., Mayo J.R., Mehta A.C., Ohno Y., Powell C.A., Prokop M. (2017). Guidelines for Management of Incidental Pulmonary Nodules Detected on CT Images: From the Fleischner Society 2017. Radiology.

[3] Martin M.D., Kanne J.P., Broderick L.S., Kazerooni E.A., Meyer C.A. (2023). *RadioGraphics* Update: Lung-RADS 2022. RadioGraphics.

[4] Dong C., Loy C.C., He K., Tang X. (2016). Image Super-Resolution Using Deep Convolutional Networks 2015. IEEE Trans. Pattern Anal. Mach. Intell..

[5] Kim J., Lee J.K., Lee K.M. Accurate Image Super-Resolution Using Very Deep Convolutional Networks. Proceedings of the 2016 IEEE Conference on Computer Vision and Pattern Recognition (CVPR).

[6] Lim B., Son S., Kim H., Nah S., Lee K.M. Enhanced Deep Residual Networks for Single Image Super-Resolution. Proceedings of the 2017 IEEE Conference on Computer Vision and Pattern Recognition Workshops (CVPRW).

[7] Zhang Y., Li K., Li K., Wang L., Zhong B., Fu Y., Ferrari V., Hebert M., Sminchisescu C., Weiss Y. (2018). Image Super-Resolution Using Very Deep Residual Channel Attention Networks. Computer Vision—ECCV 2018, Proceedings of the 15th European Conference, Munich, Germany, 8–14 September 2018.

[8] Ledig C., Theis L., Huszar F., Caballero J., Cunningham A., Acosta A., Aitken A., Tejani A., Totz J., Wang Z. Photo-Realistic Single Image Super-Resolution Using a Generative Adversarial Network 2017. Proceedings of the 2017 IEEE Conference on Computer Vision and Pattern Recognition (CVPR).

[9] Wang X., Yu K., Wu S., Gu J., Liu Y., Dong C., Qiao Y., Loy C.C., Leal-Taixé L., Roth S. (2019). ESRGAN: Enhanced Super-Resolution Generative Adversarial Networks. Computer Vision—ECCV 2018 Workshops, Proceedings of the 15th European Conference, Munich, Germany, 8–14 September 2018.

[10] Wang X., Xie L., Dong C., Shan Y. Real-ESRGAN: Training Real-World Blind Super-Resolution with Pure Synthetic Data. Proceedings of the 2021 IEEE/CVF International Conference on Computer Vision Workshops (ICCVW).

[11] Goo J.M. (2021). Deep Learning–Based Super-Resolution Algorithm: Potential in the Management of Subsolid Nodules. Radiology.

[12] Park S., Lee S.M., Kim W., Park H., Jung K.-H., Do K.-H., Seo J.B. (2021). Computer-Aided Detection of Subsolid Nodules at Chest CT: Improved Performance with Deep Learning–Based CT Section Thickness Reduction. Radiology.

[13] Park S., Lee S.M., Do K.-H., Lee J.-G., Bae W., Park H., Jung K.-H., Seo J.B. (2019). Deep Learning Algorithm for Reducing CT Slice Thickness: Effect on Reproducibility of Radiomic Features in Lung Cancer. Korean J. Radiol..

[14] Yun H.R., Lee M.J., Hong H., Shim K.W., Jeon J. (2021). Improvement of Inter-Slice Resolution Based on 2D CNN with Thin Bone Structure-Aware on Head-and-Neck CT Images. Proceedings of the Medical Imaging 2021: Image Processing.

[15] Yun H.R., Lee M.J., Hong H., Shim K.W. (2022). Inter-Slice Resolution Improvement Using Convolutional Neural Network with Orbital Bone Edge-Aware in Facial CT Images. J. Digit. Imaging.

[16] Kudo A., Kitamura Y., Li Y., Iizuka S., Simo-Serra E. Virtual Thin Slice: 3D Conditional GAN-Based Super-Resolution for CT Slice Interval. Proceedings of the Machine Learning for Medical Image Reconstruction.

[17] Nakamoto A., Hori M., Onishi H., Ota T., Fukui H., Ogawa K., Masumoto J., Kudo A., Kitamura Y., Kido S. (2022). Three-Dimensional Conditional Generative Adversarial Network-Based Virtual Thin-Slice Technique for the Morphological Evaluation of the Spine. Sci. Rep..

[18] Iwano S., Kamiya S., Ito R., Kudo A., Kitamura Y., Nakamura K., Naganawa S. (2023). Measurement of Solid Size in Early-Stage Lung Adenocarcinoma by Virtual 3D Thin-Section CT Applied Artificial Intelligence. Sci. Rep..

[19] PixelShuffle—PyTorch 2.5 Documentation. https://pytorch.org/docs/stable/generated/torch.nn.PixelShuffle.html.

[20] Johnson J., Alahi A., Fei-Fei L., Leibe B., Matas J., Sebe N., Welling M. (2016). Perceptual Losses for Real-Time Style Transfer and Super-Resolution. Computer Vision—ECCV 2016, Proceedings of the 14th European Conference, Amsterdam, The Netherlands, 11–14 October 2016.

